# Bee Bread Can Alleviate Lipid Abnormalities and Impaired Bone Morphology in Obese Zucker Diabetic Rats

**DOI:** 10.3390/molecules26092616

**Published:** 2021-04-29

**Authors:** Monika Martiniakova, Jana Blahova, Veronika Kovacova, Martina Babikova, Vladimira Mondockova, Anna Kalafova, Marcela Capcarova, Radoslav Omelka

**Affiliations:** 1 Department of Zoology and Anthropology, Faculty of Natural Sciences, Constantine the Philosopher University in Nitra, 949 74 Nitra, Slovakia; vkovacova@ukf.sk; 2 Department of Botany and Genetics, Faculty of Natural Sciences, Constantine the Philosopher University in Nitra, 949 74 Nitra, Slovakia; jana.blahova@ukf.sk (J.B.); martina.babikova@ukf.sk (M.B.); vmondockova@ukf.sk (V.M.); 3 Department of Animal Physiology, Faculty of Biotechnology and Food Sciences, Slovak University of Agriculture in Nitra, 949 76 Nitra, Slovakia; anna.kalafova@uniag.sk (A.K.); marcela.capcarova@uniag.sk (M.C.)

**Keywords:** bee bread, functional food, diabetes mellitus, lipid profile, bone morphology, ZDF rat

## Abstract

This study examined for the first time whether bee bread (BB, consisting of monofloral rape bee pollen) could alleviate lipid derangements and reduced bone quality in Zucker diabetic fatty (ZDF) rats, which are considered an appropriate animal model for type 2 diabetes mellitus (T2DM) investigation. Adult ZDF rats were segregated into four groups: lean non-diabetic rats (L group), obese diabetic rats untreated (C group), and those treated with the BB at two doses (500 and 700 mg/kg body weight, respectively, B1 and B2 groups) for 10 weeks. Significantly reduced levels of total cholesterol and triglyceride were recorded in the B2 group versus the C group. In both BB-treated groups, significantly increased relative volume of trabecular bone and trabecular thickness, enhanced density of secondary osteons, accelerated periosteal bone apposition, and improved blood flow were observed. A positive effect of higher dose of BB on femoral weight and cortical bone thickness was also demonstrated. Our results suggest a promising potential of BB to ameliorate T2DM-related complications associated with lipid and bone damages.

## 1. Introduction

Type 2 diabetes mellitus (T2DM) represents one of the most frequent public health problems with increasing prevalence worldwide. The World Health Organization states that the number of people with diabetes will double in the next ten years [1]. Generally, T2DM is a multifactorial chronic endocrine disorder [2] manifested by hyperglycaemia. This condition is caused by increased hepatic glucose production, lower insulin secretion, and impaired insulin action [3]. Subsequently, the lipid is used as an alternative resource of cellular energy, resulting in abnormalities of lipid metabolism. Hyperlipidaemia is characterised by serum lipids alterations, especially triglyceride (TG) increase and high-density lipoprotein (HDL) cholesterol decrease [4]. Another complication associated with T2DM represents diabetic bone disease. It is manifested by varied bone mineral density (BMD), abnormalities in skeletal microarchitecture, differences in bone metabolism, and lower bone strength. Chronic hyperglycaemia causes reduced expression of genes affecting the function of osteoblasts, downregulation of vitamin D receptors, enhanced bone marrow mesenchymal cells differentiation into adipocytes, increased production of advanced glycation end-products (AGEs) inhibiting bone remodelling, and higher oxidative stress [5,6]. All these conditions lead to altered bone morphology and increased risk of fractures.

Therapy for T2DM can be supported by various biological substances. Bee products are considered to be well-known functional foods. They contain a lot of proteins, sugars, essential amino acids, fatty acids, macro and microelements, and vitamins, [7] which are responsible for their high nutritional value [8]. Because of a presence of other bioactive compounds also having a beneficial effect on human health, they are useful tools for therapeutical approach as well [9]. In general, bee bread (BB), not a very well-known bee product, is the result of bee pollen fermentation in hives [10]. The bees (*Apis mellifera*) mix plant pollen with nectar or honey and their salivary enzymes [11] and fill the cells in hives with this mixture. Afterwards, anaerobic lactic fermentation begins [12] that decreases pH and enhances the bioavailability of nutrients [13,14]. Depending on geographical region, botanical origin, and storage conditions, the chemical composition of BB may differ. Salutary health impacts of BB have been rarely investigated thus far and they are conditioned by the presence of phytosterols, fatty acids, polyphenols and polysaccharides with anticancer properties, and antioxidant and immunological properties [15]. It is also regarded as a promising anti-microbial agent [7]. However, data showing the potential of BB to alleviate T2DM-reduced bone quality and hyperlipidaemia is missing. Zucker diabetic fatty (ZDF) rats are successfully used as a well-matched animal model for T2DM research [3]. They carry a spontaneous mutation in the leptin receptor gene and a defect in β-cell transcription that contributes to the diabetic phenotype [16]. ZDF rats show enhanced body weights by five weeks and their obesity gradually worsens with age [17].

This study was carried out to determine if BB could improve the diabetic lipid profile and impaired bone morphology using an in vivo animal model of obese Zucker diabetic rats. Femoral bone structure of BB-treated rats was assessed for the first time using both 3D and 2D imaging methods.

## 2. Results

### 2.1. Biochemical Analysis

**Figure 1:**
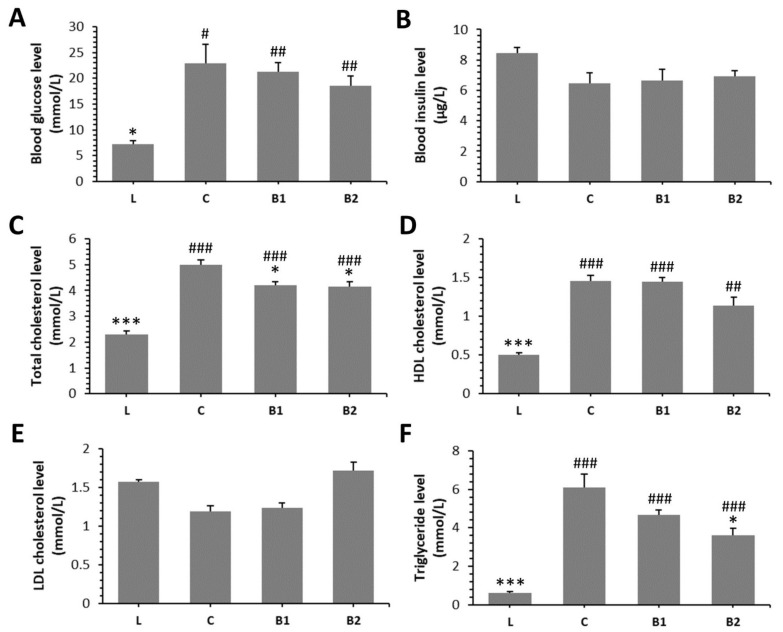
Effects of bee bread (BB) on levels of blood glucose (**A**), blood insulin (**B**), total cholesterol (**C**), high-density lipoprotein (HDL) cholesterol (**D**), low-density lipoprotein (LDL) cholesterol (**E**), triglyceride (**F**) in Zucker diabetic fatty (ZDF) rats. * Significant differences compared with C group (*p* < 0.05), *** significant differences compared with the C group (*p* < 0.001), # significant changes in relation to the L group (*p* < 0.05), ## significant changes in relation to the L group (*p* < 0.01), and ### significant changes in relation to the L group (*p* < 0.001).

The diabetic rats from all groups (C, B1, B2) had significantly elevated levels of blood glucose (BG), total cholesterol (TC), and TG as compared to lean rats (L group). A higher dose of BB (B2 group) significantly reduced TC and TG levels versus the C group. Moreover, decreased value for TC was recorded in the B1 group compared with the C group. Nonetheless, BG, blood insulin (BI), HDL cholesterol (HDLC), and low-density lipoprotein (LDL) cholesterol levels did not differ significantly in both the B1 and B2 groups versus the C group. The findings on the effect of BB on examined biochemical parameters in ZDF rats are summarized in Figure 1A–F.

### 2.2. Macroscopical Analysis of Bones

**Figure 2:**
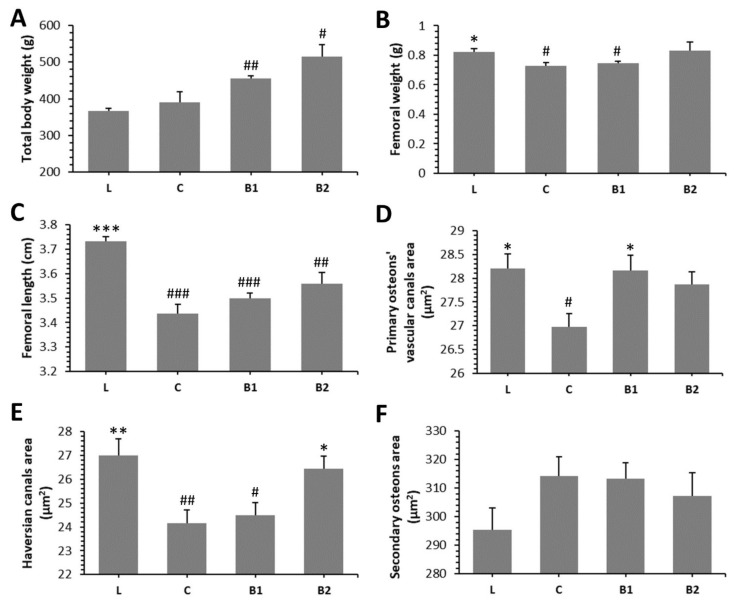
Impacts of BB on macroscopical (**A**–**C**) and morphological 2D (**D**–**F**) bone parameters in ZDF rats. * Significant differences compared with the C group (*p* < 0.05), ** significant differences compared with the C group (*p* < 0.01), *** significant differences compared with the C group (*p* < 0.001), # significant changes in relation to the L group (*p* < 0.05), ## significant changes in relation to the L group (p < 0.01), and ### significant changes in relation to L group (*p* < 0.001).

The post-treatment body weight (BW) in both BB-treated groups was higher than that determined in lean rats. Significantly decreased femoral weight (FW) was observed in the C and B1 groups versus the L group. No significant difference in FW between L and B2 groups reflects a beneficial effect of a higher dose of BB on this parameter. However, total BW, femoral length (FL), and FW were non-significantly higher in the B1 and B2 groups as compared to the C group. The results are presented in Figure 2A–C.

### 2.3. Morphological 3D and 2D Analyses of Bones

**Figure 3:**
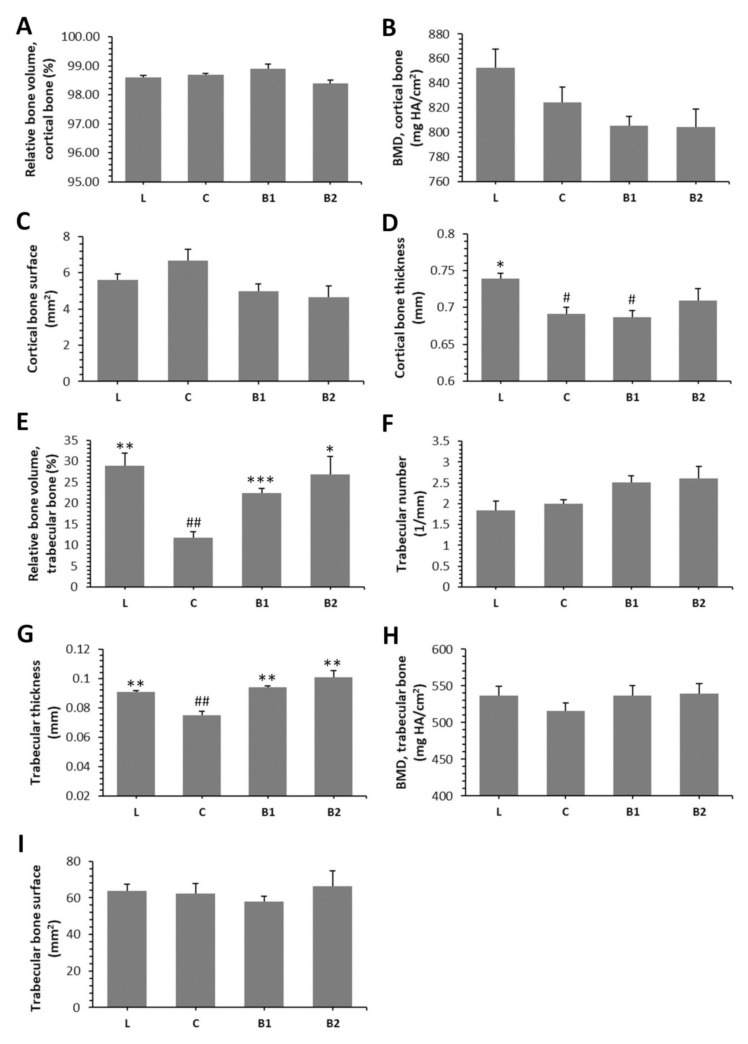
Effects of BB on morphological 3D parameters of the cortical bone (**A**–**D**) and trabecular bone (**E**–**I**) in ZDF rats. BMD—bone mineral density. * Significant differences compared with the C group (*p* < 0.05), ** significant differences compared with the C group (*p* < 0.01), *** significant differences compared with the C group (*p* < 0.001), # significant changes in relation to the L group (*p* < 0.05), and ## significant changes in relation to L group (*p* < 0.01).

**Figure 4:**
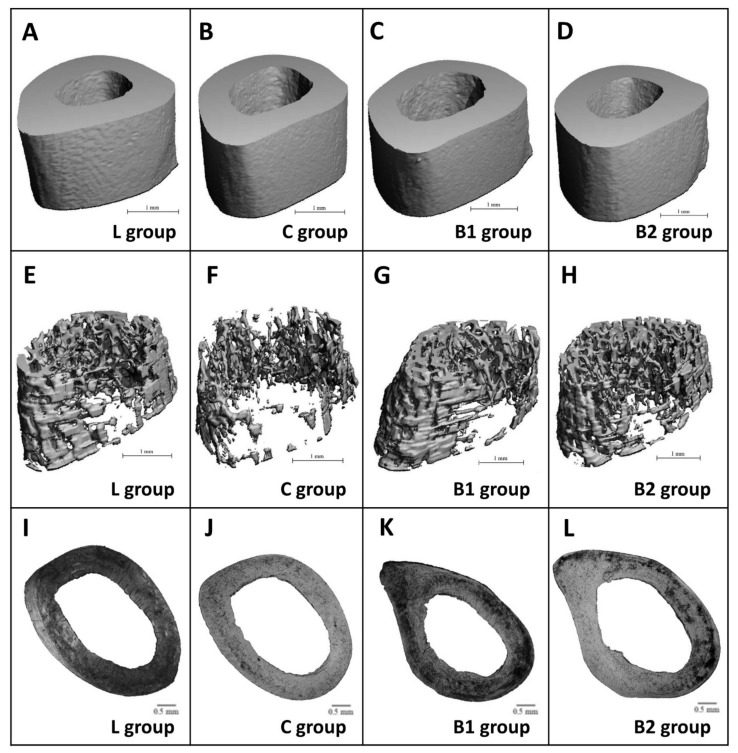
Representative 3D images of cortical (**A**–**D**) and trabecular bone tissues (**E**–**H**), and representative 2D images of the cortical bone (**I**–**L**) in ZDF rats.

Treatment with BB had an insignificant effect on relative bone volume (BV/TV) of cortical bone, cortical bone surface (BS), and BMD. Despite this, significantly decreased cortical bone thickness (Ct.Th.) was recorded in the C and B1 groups versus the L group. No significant changes between L and B2 groups insinuate a positive impact of a higher dose of BB on Ct.Th. The results are provided in Figure 3A–D. Figure 4A–D shows representative reconstructed 3D images of the cortical bone. Compared with diabetic control rats, rats from both the B1 and B2 groups had significantly elevated relative bone volume (BV/TV) of the trabecular bone and trabecular thickness (Tb.Th.). No significant impact of BB administration on trabecular number (Tb.N.), BMD, and trabecular bone surface (BS) was recorded. The data are shown in Figure 3E–I. Figure 4E–H illustrates representative reconstructed 3D images of the trabecular bone.

In general, non-vascular bone tissue formed both surfaces of the cortical bone in all groups studied. Near the endosteal surface and in the middle of substantia compacta, primary vascular radial bone tissue was observed. Several secondary osteons (SO) have also been identified in the middle part of the cortical bone. We recorded a higher density of SO and accelerated periosteal bone apposition in both BB-treated groups versus the C group (Figure 4I–L). Significantly higher areas of primary osteons’ vascular canals (POVC) were established in the B1 and L groups versus the C group. Compared with diabetic control rats, these from the B2 and L groups had significantly elevated areas of Haversian canals (HC). On the other hand, treatment with BB did not influence SO area. The data are summarized in Figure 2D–F.

## 3. Discussion

Bee products have been used as alternative medicine to treat various serious disorders including diabetes mellitus [18]. Our study, for the first time, examined the protective impacts of BB (formed by monofloral rape bee pollen) against T2DM-related complications consistent with lipid and bone disorders. Therefore, comparison with other studies using either identical bee product, animal model, or both, was not possible in this field of research. Only our findings related to BG, BI levels, and total BW of ZDF rats were discussed with published researches focused on BB and obese Zucker diabetic rats.

Capcarova et al. [19] state that ZDF rats receiving BB at a dose of 700 mg/kg BW (for 10 weeks) had significantly lower BG levels only in the pre-diabetic state versus the diabetic control group. At the end of the treatment, BG did not change significantly between treated and control groups, thus identical to our findings. Nevertheless, aforementioned decreases of BG during the experiment might also influence some biochemical and morphological parameters as documented in our study. We recorded non-significant reduction of BG levels in both B1 and B2 groups. Generally, lower BG levels and anti-diabetic activities of BB could be consistent with the presence of polyphenol compounds [20]. Among polyphenols, flavonoids represent the largest group. It is known that differences in flavonoid fraction may be conditioned by geographic profile. According to Tavdidishvili et al. [21], the main flavonoids were naringenin, rutin, and quercetin in Georgian BB samples. Sobral et al. [22] identified quercetin, myricetin, isorhamnetin, kaempferol, and herbacetrin glycoside derivatives as the most abundant phenolic compounds in BB samples from Portugal. In BB specimens from Romania, kaempferol, myricetin, and luteolin as the most numerous polyphenols were determined [23]. Similarly, Markiewicz-Zukowska et al. [24] reported kaempferol and apigenin as the main flavonoids in Polish BB. In BB samples from Ukraine, kaempferol, quercetin, apigenin, and naringenin were established [25]. According to Zhang et al. [26], major flavonoids in rape bee pollen ethanol extract included quercetin, kaempferol, and rutin. Onuekwuzu et al. [27] revealed that treatment with flavonoid and sitosterol-rich aqueous extract had hypoglycaemic effect and improved lipid abnormalities in alloxan-induced diabetic rats. Jung et al. [28] determined flavonoid derivates (also present in BB) as potential inhibitors of AGEs because glycation can lead to the onset of diabetic complications due to chronic hyperglycaemia.

In the study by Capcarova et al. [19], treatment with BB (700 mg/kg BW for 10 weeks) non-significantly increased BI levels in ZDF rats in contrast to the diabetic control group, which is in line with our findings. Since many flavonoids (including quercetin) found in BB scavenge reactive oxygen species (ROS) and might protect β cells from oxidative damage, they could also increase the circulating BI level. To support this hypothesis, quercetin (15 mg/kg/d intraperitoneally injected for 4 weeks) was determine to partially restore the streptozotocin (STZ)-induced insulin deficiency in rats [29].

Enhanced levels of TG, TC, HDLC, and LDL cholesterol (LDLC) were recorded in our diabetic control group versus the lean one, thus supporting the findings of Pang et al. [30] and Zhou et al. [31]. Our results revealed that a higher dose of BB reduces TG and TC levels, presumably due to hypotriglyceridaemic and hypocholesterolemic properties of BB. Significantly decreased TC and TG values were also observed in alloxan-induced diabetic rats supplemented with Nigerian honey (1.0 or 2.0 g/kg for 3 weeks) [32].

BB supplementation insignificantly increased total BW of ZDF rats in our study. The decrease in BW that occurs involuntarily, was set as a warning sign of diabetes. Although no significant differences in total BW were determined in B1 and B2 groups versus the C one, there is a tendency for higher BW in both treated groups. Capcarova et al. [19] also noted a negligible increase in post-treatment BW of ZDF rats receiving the same dose of BB (700 mg/kg BW for 10 weeks). In the study by Zaid et al. [33], no significant differences in total BW of ovariectomised (OVX) rats following Tualang honey administration were also reported. However, it is necessary to state that BB is richer in protein (up to 40%) than honey (about 0.5%). Similarly, treatment with bee pollen (0.2% for 90 d) had insignificant effect on total BW, FW, and FL of Wistar rats [34].

According to 3D imaging, supplementation with BB had more positive effects on the trabecular versus cortical bone morphology in ZDF rats. Zaid et al. [33] reported significant increase in BV/TV of trabecular bone, Tb.Th., and Tb.N. in OVX rats treated with Tualang honey (0.2 g/kg BW for 6 weeks). In addition, these rats showed more improvements in trabecular bone microarchitecture than the rats receiving 1% calcium in their drinking water. According to Tomaszewska et al. [35], bee pollen supplementation (10 g/kg of feed) enlarged trabecular BV/TV and maximal Tb.Th. in Japanese quails as well. The results by Yamaguchi et al. [36] reflect that bee pollen administration increases calcium content in femoral bones of rats, thus showing a beneficial impact on mineralization of the trabecular bone. In relation to the cortical bone, supplementation with *Apis dorsata* honey (2 g/kg BW for 3 months) can inhibit reduced Ct.Th. in OVX rats [37]. We also identified slightly improved Ct.Th. in the B2 group.

Overall, different effects of BB administration on the cortical and trabecular bone structure may be associated with their unequal levels of bone remodelling. The trabecular bone has a large area exposed to bone marrow and blood flow, and bone turnover is greater there. In the cortical bone, 2D imaging revealed accelerated periosteal bone formation and larger density of SO in both BB-treated groups. Generally, periosteal bone apposition represents an adaptive response to maintain bone strength during aging, while bone is resorbed in the sub-endocortical envelope [38]. The amount of periosteal apposition required to maintain bone strength during aging depends on adult bone morphology and tissue-modulus degradation rate. Furthermore, it is commonly known that enhanced density of smaller SO decelerates microdamage propagation [39] and is consistent with greater bone strength. Our results suggest that treatment with BB improved cortical bone strength via both aforementioned mechanisms.

We determined significantly increased sizes of POVC and HC in the B1 and B2 groups, respectively, versus the C group. This evidence could be associated with a vasodilation of blood vessels in both canals. According to Peng et al. [40], T2DM leads to the development of microangiopathy which is consistent with a vasoconstriction of blood vessels. Actually, Stabley et al. [41] identified vasoconstriction and impaired blood flow in long bones of ZDF rats. Peng et al. [42] revealed a reduced angiogenesis accompanied by a lower number of blood vessels in the femurs of STZ-induced diabetic mice. Generally, blood vessels within the POVC and HC pass above the medullary cavity, and through the trabeculae of the trabecular bone, thus providing oxygen, nutrients, hormones, or growth factors for both cortical and trabecular bone tissues [43]. In agreement with our results, BB supplementation had a beneficial effect on blood flow in the femoral bones of ZDF rats.

## 4. Materials and Methods

### 4.1. Ethics Statement

All experimental procedures were authorized under the number 2288/16-221 by the Ethical Committee and the State Veterinary and Food Institute of the Slovak Republic. Institutional and national guidelines for the care and use of animals, including EU Directive 2010/63/EU for animal experiments, were adequately followed.

### 4.2. Sample Preparation

**Figure 5:**
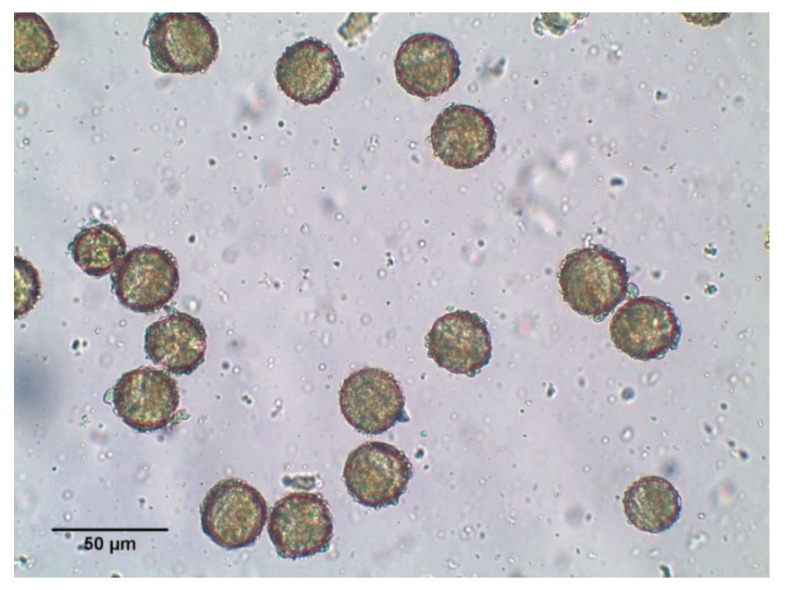
Microphotography of BB used in our experiment.

The BB was provided by the Institute of Biodiversity Conservation and Biosafety of the Slovak University of Agriculture in Nitra (Slovak Republic). It consisted of monofloral rape (*Brassica napus L.*) bee pollen (Figure 5). Table 1 shows a chemical composition of BB used in our experiment. No toxic substances including heavy metals, pesticides, and mycotoxins were detected. BB was crushed, mixed with distilled water, and homogenized to be convenient for application by gastric gavage.

### 4.3. Animals

Adult male ZDF rats (*n* = 32) came from the breeding station of the Slovak Academy of Sciences and were bred at the Slovak University of Agriculture in Nitra (Slovakia). All animals were housed in pairs under standardized conditions with 12:12 h light-dark cycles and temperature of 22 ± 2 °C. They were fed a complete feed mixture for rats on an ad libitum basis and were assigned into 4 groups of 8 individuals each: lean (L group) consisted of non-diabetic rats, control (C group) included diabetic obese rats, and groups B1 and B2 consisted of ZDF treated with the BB at doses of 500 and 700 mg/kg BW, respectively, for 10 weeks. The exact doses of BB in both the B1 and B2 groups were applied directly into stomach by oral rodent gavage every day, whilst L and C groups received distilled water. The doses used were chosen according to the study by Capcarova et al. [19].

### 4.4. Biochemical Analysis

ZDF rats were sacrificed by intraperitoneal anaesthetic overdose of xylazine/zoletil mixture. Whole blood was processed and the levels of investigated parameters (BG, BI, TC, LDLC, HDLC, and TG) were subsequently examined. Table 2 illustrates all measured parameters and appropriate methods which were used to obtain them.

### 4.5. Macroscopical Analysis of Bones

BW and BL of both femurs (*n* = 64) were measured. Furthermore, total BW of all ZDF rats was recorded.

### 4.6. Morphological 3D and 2D Analyses of Bones

Microstructural 3D analyses of femoral bones were performed by 3D imaging (micro-CT 50, Scanco Medical, Brüttisellen, Switzerland). Cortical bone structure was assessed in the region of interest (ROI) starting 12.5 mm from the end of the growth plate (distal end) and ending 2.0 mm. Evaluated parameters of the cortical bone (BV/TV, BMD, Ct.Th., and BS) are included in Table 2. Trabecular bone structure was analysed in the ROI starting 2.9 mm from the end of the growth plate and exceeding 2.0 mm. Examined parameters of the trabecular bone (BV/TV, Tb.N., Tb.Th., BMD, and BS) are shown in Table 2. High resolution scans with 14.8 µm voxel size were obtained in both ROIs, including scanning parameters 70 kVp, 200 mA, 300 ms, and 0.5 mm aluminium filter [44]. Thin sections from cortical bone tissue (2D imaging) were prepared according to Martiniakova et al. [45] and were evaluated by established classification systems [46,47]. Measured parameters included area of POVC (*n* = 796), HC (*n* = 329), and SO (*n* = 329). They were determined by Motic Images Plus 2.0 ML software (Motic China Group Co., Ltd., Nanjing, China).

### 4.7. Statistical Analysis

Statistical analysis was conducted using SPSS Statistics 26.0 software. The data were expressed as mean ± standard error of the mean. Differences in the parameters examined were detected by ANOVA with either Games–Howell’s, Tukey’s, or both, post hoc tests. Statistical significance was assessed at *p* < 0.05, *p* < 0.01, and *p* < 0.001.

## 5. Conclusions

Our study revealed that treatment with BB has a possible potential to improve lipid disorders and reduced bone morphology in ZDF rats. Therefore, it can be used as a nutritional supplement to ameliorate T2DM-related complications associated with hyperlipidaemia and diabetic bone disease. However, determination of the optimal dose and duration of the BB-therapy requires further experiments.

## Figures and Tables

**Table 1:** Chemical composition of BB used in the experiment.

Group (Units)	Item	Content	Method
Main components (g/100 g)	Dry matter	71.35	GA
	Protein	19.27	KM
	Fat	6.48	GA
	Carbohydrates	2.09	CAL
	Fibre	41.25	EGM
Macroelements (g/kg)	Ca	1.50	ICP-AES
	Mg	0.91	ICP-AES
	P	4.48	ICP-AES
	K	4.55	ICP-AES
	Na	0.06	ICP-AES
Microelements (mg/kg)	Cu	5.90	F-AAS
	Se	0.21	HG-AAS
	Cr	0.14	ETA-AAS
	Ni	0.77	ETA-AAS
	Co	<0.10	ETA-AAS
Vitamins (mg/kg)	A (retinol acetate)	<0.05	HPLC-DAD
	E (α-tocopherol acetate)	28.4	HPLC-DAD
	C (ascorbic acid)	<1.0	HPLC-DAD
	B2 (riboflavin)	9.6	HPLC-DAD
	B3 (PP; nicotinamide)	11.5	HPLC-DAD
	Beta carotene	16.5	UV-VIS
Fatty acids (g/100 g)	Saturated fatty acids	3.91	GC-FID
	Monounsaturated fatty acids	0.76	GC-FID
	Polyunsaturated fatty acids	0.81	GC-FID
Amino acids (g/kg)	Asp	19.0	IEC
	Thr	7.7	IEC
	Ser	7.1	IEC
	Glu	21.7	IEC
	Pro	15.0	IEC
	Gly	9.6	IEC
	Ala	9.6	IEC
	Val	10.5	IEC
	Ile	8.9	IEC
	Leu	13.7	IEC
	Lys	11.3	IEC
	Arg	7.8	IEC
	Tyr	4.3	IEC
	Phe	10.5	IEC
	His	5.2	IEC
	Cys	8.2	IEC
	Met	11.9	IEC
	Trp	2.2	IEC

GA—gravimetric analysis; KM—Kjeldahl method; EGM—enzymatic-gravimetric method using Total Dietary Fiber Assay Kit (Megazyme); CAL—calculations from polarimetric, spectrophotometric, and volumetric analyses; ICP-AES—inductively coupled plasma atomic emission spectroscopy; F-AAS—flame atomic absorption spectroscopy; ETA-AAS—electrothermal atomization atomic absorption spectrometry; HG-AAS—hydride generation atomic absorption spectroscopy; HPLC-DAD—high-performance liquid chromatography with a diode-array detector; UV-VIS—ultraviolet-visible spectroscopy; GC-FID—gas chromatography with flame-ionization detection; and IEC—ion exchange chromatography. The analyses were performed in accordance with Slovak technical standards by an accredited laboratory EL Ltd (Spisska Nova Ves, Slovakia).

**Table 2:** Measured parameters from biochemical and morphological 3D and 2D analyses.

Type of Analysis	Parameter	Unit	Method
Biochemistry	blood glucose (BG)	mmol/L	POC glucometer ^1^
	blood insulin (BI)	μg/L	ELISA ^2^
	total cholesterol (TC)	mmol/L	colorimetric ^3^
	LDL cholesterol (LDLC)	mmol/L	colorimetric ^3^
	HDL cholesterol (HDLC)	mmol/L	colorimetric ^3^
	triglycerides (TG)	mmol/L	colorimetric ^3^
Cortical bone—3D imaging	relative bone volume (BV/TV)	%	micro-CT
	bone mineral density (BMD)	mg HA/ccm	micro-CT
	cortical bone thickness (Ct.Th.)	mm	micro-CT
	bone surface (BS)	mm^2^	micro-CT
Cortical bone—2D imaging	area of primary osteons’ vascular canals (POVC)	μm^2^	histomorphometry
	area of Haversian canals (HC)	μm^2^	histomorphometry
	area of secondary osteons (SO)	μm^2^	histomorphometry
Trabecular bone—3D imaging	relative bone volume (BV/TV)	%	micro-CT
	trabecular number (Tb.N.)	1/mm	micro-CT
	trabecular thickness (Tb.Th.)	mm	micro-CT
	bone mineral density (BMD)	mg HA/ccm	micro-CT
	bone surface (BS)	mm^2^	micro-CT

^1^ FreeStyle Optium Neo Blood Glucose and Ketone Monitoring System (Abbott, Alameda, USA); ^2^ Ultrasensitive Rat Insulin ELISA (Mercodia AB, Uppsala, Sweden); ^3^ commercially available kits (Randox Laboratories Ltd, Crumlin, UK) analysed by Biolis 24i Premium biochemical analyser (Tokyo Boeki MediSys Inc., Japan).

## Data Availability

Data is contained within the article.

## Sample Availability

Samples of the compounds are available from the authors.
